# Recurrent Cholesteatoma with Skull Base Erosion: A Case Report

**DOI:** 10.7759/cureus.79460

**Published:** 2025-02-22

**Authors:** Abdulrahman Alosaimi, Ibrahim A Tawfiq, Naeem Makhdoom

**Affiliations:** 1 Otolaryngology-Head and Neck Surgery, Ohud Hospital, Madinah, SAU; 2 Medicine, Taibah University, Madinah, SAU; 3 Otolaryngology-Head and Neck Surgery, Taibah University, Madinah, SAU

**Keywords:** cholesteatoma surgery, complications of acute mastoiditis, reconstruction of skull base, temporal bone imaging, tympanomastoidectomy

## Abstract

Cholesteatomas are non-malignant, expandable diseases that, if left untreated, can destroy the middle ear, temporal bone, and other structures. Their recurrence can cause disastrous effects, for example, skull base destruction, acute mastoiditis, and brain abscess, etc. Therefore, they need early diagnosis, and it might necessitate surgical intervention according to the complications.

We present the case of a 30-year-old male patient who came to the emergency department with a picture of acute right-sided mastoiditis. The patient complained of a one-day history of right ear pain, ear swelling, fever, and purulent-bloody right-sided ear discharge. On examination, there was a postauricular swelling with redness and a whitish, thick, offensive secretion coming out of the stenosed external auditory canal (EAC) as well as a white mass in the stenosed EAC. The patient had ear surgery 14 years ago, with recurrence of the cholesteatomas, acute mastoiditis, and skull base erosion. The pure tone audiogram showed a big right-sided conductive hearing loss of 35 dB air-bone gap. Mastoid and temporal bone destruction, skull base defects, and jugular vein encasement were noted on CT, MRI, and brain angiogram of the patient. The patient underwent a revision tympanomastoidectomy with skull base reconstruction.

This case report underscores the significance of early diagnosis, adequate imaging, and appropriate management of the disease with possible complications, including acute mastoiditis and skull base infiltration, which will help prevent the development of dangerous consequences, such as cerebrospinal fluid (CSF) leaks and intracranial infections.

## Introduction

Cholesteatomas are benign, locally invasive lesions that typically form in the middle ear or mastoid cavity. They are often associated with chronic otitis media (COM) or can develop as a complication following tympanoplasty procedures [1]. Cholesteatomas are characterized by the accumulation of keratinized squamous epithelium in the middle ear, leading to the destruction of the ossicles, mastoid bone, and, in some cases, the skull base [2].

These lesions present with various symptoms, including hearing loss, persistent otorrhea, and ear fullness. In more severe cases, cholesteatomas can lead to facial nerve paralysis, intracranial infections, and skull base erosion [3]. Such recurrence is a well-known but rare occurrence after tympanomastoidectomy with serious consequences like this case, and if it happens, it can lead to severe morbidity. Such recurrences are often associated with complications like acute mastoiditis and extracranial or intracranial complications, which need proper imaging and surgical treatment for their management [4].

This case report demonstrates the clinical features, diagnostic studies, surgical procedure, and postoperative course of a patient with chronic ear disease, including recurrent cholesteatoma with acute mastoiditis and skull base erosion, thus stressing the need for early diagnosis and multimodality therapy.

## Case presentation

A 30-year-old male patient presented with a one-day history of right ear pain, purulent-bloody discharge, fever, hearing loss, and a protruding white mass from the external auditory canal (EAC). The patient had undergone ear surgery 14 years ago and had been followed for recurrent cholesteatomas. He reported intermittent episodes of otorrhea and hearing loss over the years. However, there was no history of vertigo, tinnitus, or facial nerve paralysis.

Upon examination, a postauricular swelling with redness and a whitish, thick, offensive secretion was coming out of the stenosed external auditory canal, as well as a white mass in the stenosed external auditory canal, and the tympanic membrane was not visible. There was also preauricular tenderness and bloody discharge was noted from the external auditory canal. No facial nerve paralysis was observed. While the left ear had normal thresholds, pure tone audiometry found severe conductive hearing loss in the right ear with notable air-bone gaps. The Weber test lateralized to the right, verifying conductive hearing loss; the Rinne test was negative on the right ear and positive on the left.

Preoperative workup

Some of the laboratory investigations indicated an active infection by measuring the white blood cell (WBC) and C-reactive protein (CRP), which was high, and the erythrocyte sedimentation rate (ESR), which was slightly elevated. The culture and sensitivity report showed *Pseudomonas aeruginosa* as the causative organism resistant to many antibiotics; hence, the antibiotics were given intravenously after consultation with the infectious disease team.

**Figure 1:**
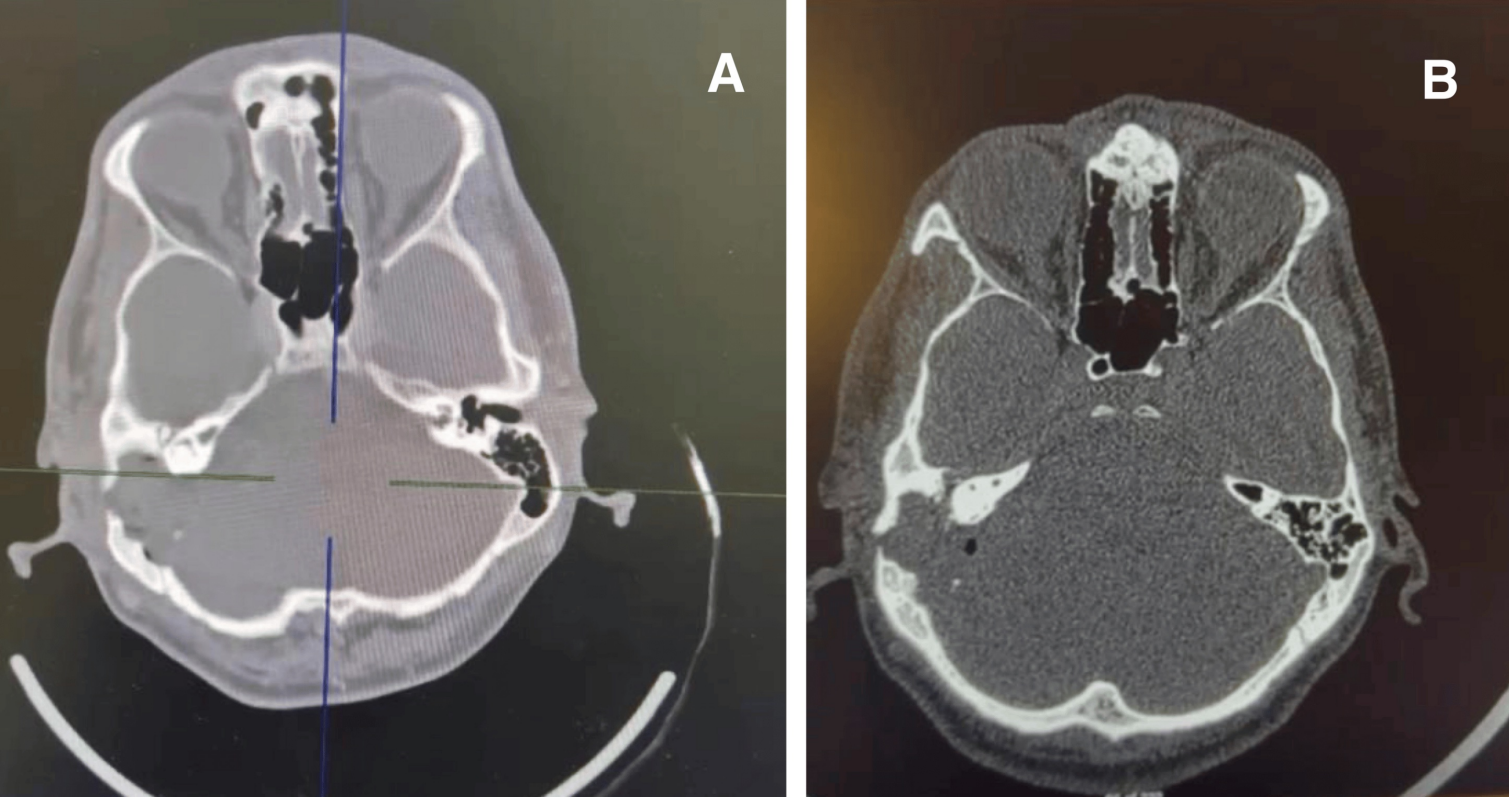
The preoperative CT scan of the right temporal bone shows a large soft tissue mass in the mastoid cavity with associated bony erosion and a focal defect in the tegmen mastoideum. A: bone window (axial cut); B: soft tissue window (axial cut)

Imaging studies revealed the following findings: A CT temporal bone scan showed a massive heterogeneously enhancing soft tissue mass in the right mastoid cavity with aggressive bone destruction and remodeling. There was a defect in the tegmen mastoideum's bone, and the tegmen tympani's bone was paper thin (Figure 1).

**Figure 2:**
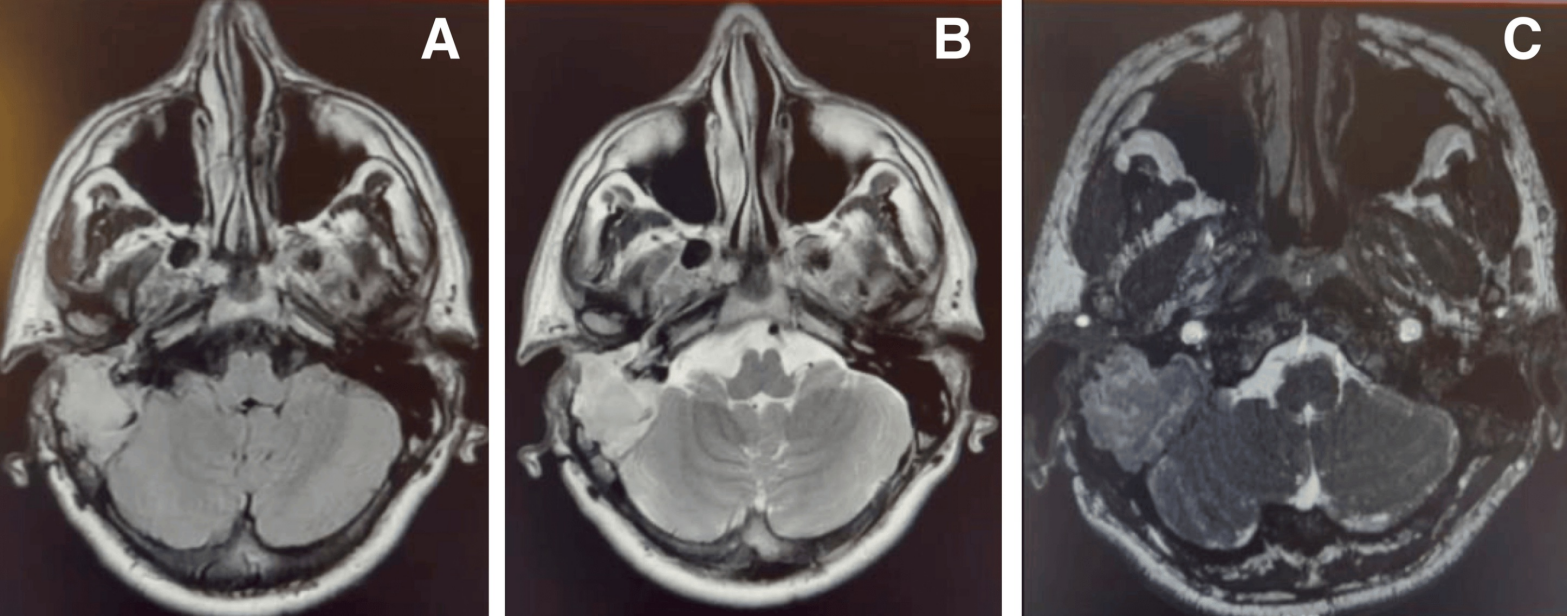
The preoperative MRI shows soft tissue intensity of the right mastoid air cells with encasement of the distal jugular vein and thinning of the tegmentum tympani. A: T1 without contrast; B: T2 without contrast; C: T1 with contrast

An MRI of the brain and internal auditory meatus (IAM) revealed soft tissue intensity of the right mastoid air cells with encasement of the distal jugular vein, causing narrowing. No enhancement or diffusion restriction was noted in the cerebellopontine angle (Figure 2).

**Figure 3:**
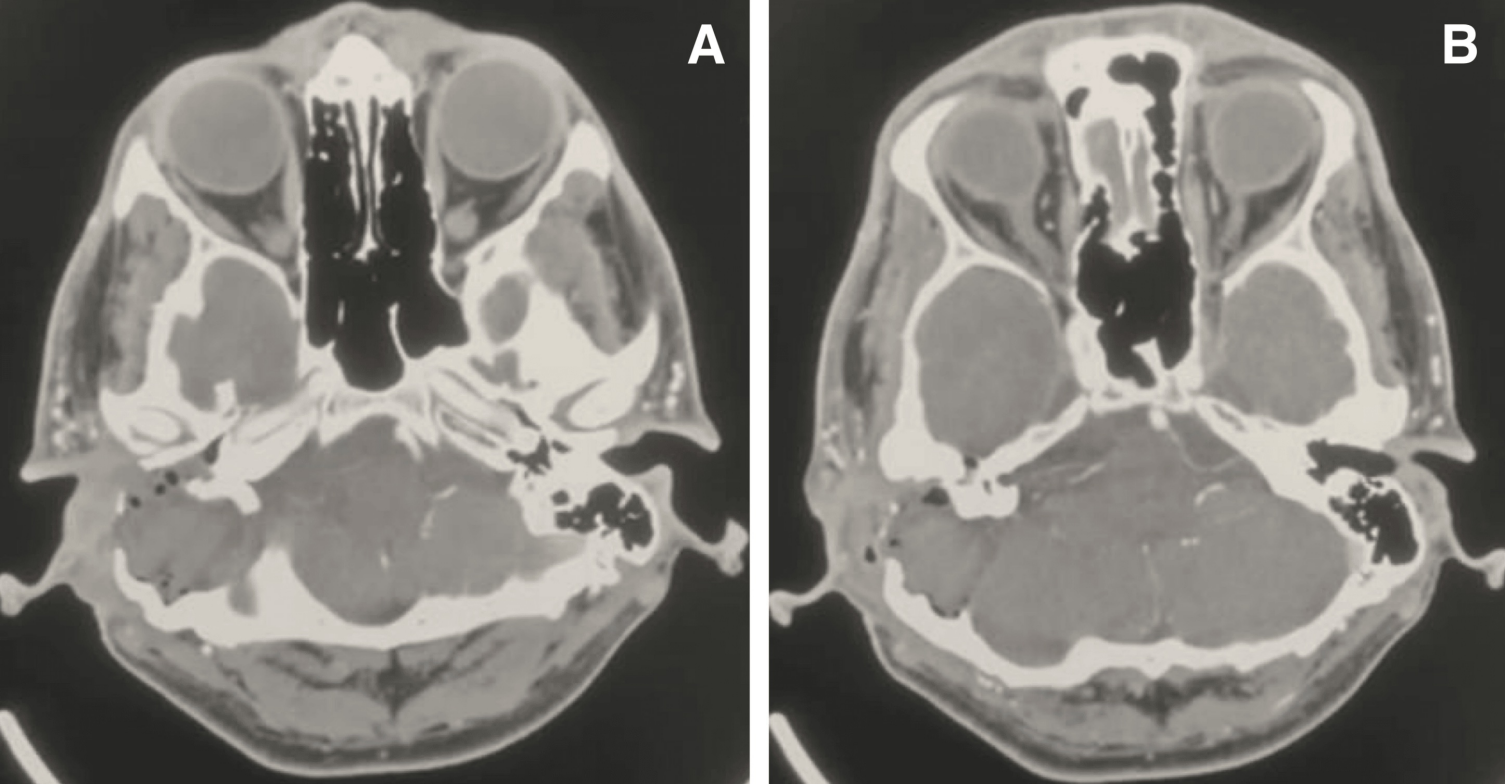
Brain angiography demonstrates destructive involvement of the external and middle ear cavities, mastoid air cells, and jugular foramen, with encasement of the internal jugular vein and narrowing extending to the sigmoid sinus. A and B: multiaxial CT cerebral angiogram

A brain angiography using a multiaxial CT cerebral angiogram also demonstrated destructive involvement of the right external and middle ear cavities, mastoid air cells, jugular foramen, and associated bony expansion and erosion. The internal jugular vein was coated, showing significant narrowing and loss of contrast in the sigmoid sinus. The remaining cerebral vasculature was unremarkable, and there was no arterial thrombus or critical stenosis, as shown in Figure 3. A neurosurgical consultation was performed, and it was agreed that the neurosurgery team should be involved in the same setting to manage the skull base erosion and optimize the management of the patient.

Procedure

The patient underwent a radical mastoidectomy with ossiculoplasty and reconstruction of the skull base defect. The operation was done under general anesthesia with local infiltration of the right ear canal using lidocaine with epinephrine (1:200,000) for hemostatic effect. Facial nerve monitors connected on the right side were tested. A postauricular incision was done, and the tympanomeatal flap was elevated. The external auditory canal was cleaned of granulation tissue and cholesteatoma, and the dissection showed that the canal was extensively involved by cholesteatoma, which had destroyed the posterior wall of the canal. Cholesteatoma was seen in the middle ear and epitympanum, and the incus and stapes superstructure were eroded.

A radical mastoidectomy was done, and the cholesteatoma was removed comprehensively to avoid touching the exposed dura. The medial wall of the mastoid cavity was found to be eroded by the cholesteatoma, necessitating reconstruction. The entire cholesteatoma from the mastoid cavity was excised and sent to histopathology, including the epitympanum, residual incus, and malleus head.

A cartilage graft from the right ear concha and perichondrium was harvested and used to reconstruct the ossicular chain with an underlay technique. A piece of cartilage was also put in between the stapes footplate and the malleus handle, and the ossicular chain was tested by applying pressure to the cartilage and ensuring it was moving. The medial wall bone defect was filled with bone cement and sealed with a piece of fascia lata. Thus, the bone cement and the fascia lata graft provided a good fixation of the defect. Meatoplasty was done, and then gel foam was applied to ensure that bleeding was controlled. A silastic sheet was positioned on the lateral aspect of the tympanic membrane wall. The postauricular incision was closed in layers.

The patient had no complaints during the surgery and was moved to the recovery unit with stable hemodynamics. The histopathology report two days after the operation confirmed the presence of cholesteatoma and excluded malignancy.

## Discussion

Cholesteatomas are characterized by their invasive nature, which results in several consequences, including hearing loss, facial nerve palsy, and extracranial or intracranial complications [5]. The recurrence of cholesteatomas after tympanoplasty is not very common. Still, it is a serious complication as it may cause acute mastoiditis, skull base erosion, and even damage to vital structures like the internal jugular vein, sigmoid sinus, or even carotid artery [4, 3]. This case report shows how recurrent cholesteatoma, accompanied by acute mastoiditis, can cause severe bone and soft tissue erosion as well as skull base invasion.

Imaging is crucial in assessing and treating recurrent cholesteatomas. In this case, CT and MRI scans helped assess the defect's anatomy and the surrounding structures, including the ossicles, mastoid air cells, and the skull base [6,7]. Furthermore, brain angiography helped evaluate vascular involvement, which is crucial in determining the risk of intracranial extension and vascular impairment [8, 9]. These advanced imaging techniques are essential in formulating the treatment plan and directing surgical procedures.

The treatment of recurrent cholesteatoma with skull-base-handled complications presents a lot of challenges, of which a multidisciplinary team needs attention. In this patient, the cholesteatoma was eradicated, and the skull base defect was sealed with bone cement and fascia lata to avoid other complications like cerebrospinal fluid (CSF) leaks or intracranial infections. [10]. The use of bone cement and facial lata for skull base reconstruction has also been described, and it helps to support the structure of the skull base following the extraction of cholesteatoma to prevent future complications. [11].

Furthermore, the presence of multiple prominent cervical lymph nodes, as observed in this case, raised the concern for possible malignancy. However, a histopathological examination of the excised cholesteatoma confirmed it to be benign, and no malignancy was found. At the same time, lymphadenopathy is often associated with chronic infections; its presence warrants close monitoring and further investigation to rule out other underlying causes. This case emphasizes the need for histopathology to determine the benign nature of cholesteatoma and exclude malignancy, particularly when lymph node involvement is noted [12].

This requires a long-term follow-up to identify any possible recurrence and assess for other possible sequelae, such as worsening hearing loss or patient complications related to having to undergo base surgery. Its cholesteatoma removal must be followed up periodically so that if there is a recurrence, it is detected as soon as possible and managed appropriately [2]. This is a case where a multidisciplinary team, including otolaryngologists, neurosurgeons, and radiologists, has played an essential role in the management of the patient and thus led to the best possible results.

## Conclusions

Cholesteatomas, though non-cancerous, can cause serious consequences due to their tendency to recur and cause e, especially after tympanomastoidectomy. In this case, the recurrence of cholesteatoma, along with acute mastoiditis and skull base erosion, presented complex management issues. The surgical team used CT, MRI, and brain angiography to plan for eradicating the cholesteatoma and the reconstruction of the skull base. The histopathological assessment of the multiple lymph nodes in the cervical region revealed them to be benign. This case highlights the importance of early disease recognition, thorough investigation, and team-based management for cholesteatomas with skull base invasion. There is a need for frequent follow-up to identify any recurrence or new complications.
